# Inherited Variants in *BLM* and the Risk and Clinical Characteristics of Breast Cancer

**DOI:** 10.3390/cancers11101548

**Published:** 2019-10-13

**Authors:** Wojciech Kluźniak, Dominika Wokołorczyk, Bogna Rusak, Tomasz Huzarski, Aniruddh Kashyap, Klaudia Stempa, Helena Rudnicka, Anna Jakubowska, Marek Szwiec, Sylwia Morawska, Katarzyna Gliniewicz, Karina Mordak, Małgorzata Stawicka, Joanna Jarkiewicz-Tretyn, Magdalena Cechowska, Paweł Domagała, Tadeusz Dębniak, Marcin Lener, Jacek Gronwald, Jan Lubiński, Steven A. Narod, Mohammad R. Akbari, Cezary Cybulski, the Polish Hereditary Breast Cancer Consortium

**Affiliations:** 1 International Hereditary Cancer Center, Department of Genetics and Pathology, Pomeranian Medical University in Szczecin, 71-252 Szczecin, Poland; kluzniak.w@gmail.com (W.K.); dominikawok@o2.pl (D.W.); b_rusak@yahoo.com (B.R.); huzarski@pum.edu.pl (T.H.); kashyap@pum.edu.pl (A.K.); kastempa@gmail.com (K.S.); helena.rudnicka@pum.edu.pl (H.R.); aniaj@pum.edu.pl (A.J.); sylmoraw@gmail.com (S.M.); katarzynagliniewicz@gmail.com (K.G.); Karina_m@wp.pl (K.M.); debniak@sci.pum.edu.pl (T.D.); marcinlener@poczta.onet.pl (M.L.); jgron@pum.edu.pl (J.G.); lubinski@pum.edu.pl (J.L.); 2 Department of Clinical Genetics and Pathology, University of Zielona Góra, 65-046 Zielona Góra, Poland; gosiastawicka@wp.pl; 3 Independent Laboratory of Molecular Biology and Genetic Diagnostics, Pomeranian Medical University in Szczecin, 71-252 Szczecin, Poland; 4 Department of Surgery and Oncology, University of Zielona Góra, 65-046 Zielona Góra, Poland; szwiec72@gmail.com; 5 Cancer Genetics Laboratory, 87-100 Toruń, Poland; jarkiewicztretyn@poczta.onet.pl (J.J.-T.); mcechowska@gmail.com (M.C.); 6 Department of Pathology, Pomeranian Medical University in Szczecin, 71-252 Szczecin, Poland; paweldom@hotmail.com; 7 Women’s College Research Institute, Women’s College Hospital, Toronto, M5S 1B2 ON, Canada; Steven.Narod@wchospital.ca (S.A.N.); mohammad.akbari@utoronto.ca (M.R.A.); 8 Dalla Lana School of Public Health, University of Toronto, Toronto, M5T 3M7 ON, Canada

**Keywords:** *BLM*, mutation, cancer, breast cancer, risk, hereditary, survival

## Abstract

Bloom Syndrome is a rare recessive disease which includes a susceptibility to various cancers. It is caused by homozygous mutations of the *BLM* gene. To investigate whether heterozygous carriers of a *BLM* mutation are predisposed to breast cancer, we sequenced *BLM* in 617 patients from Polish families with a strong family history of breast cancer. We detected a founder mutation (c.1642C>T, p.Gln548Ter) in 3 of the 617 breast cancer patients (0.49%) who were sequenced. Then, we genotyped 14,804 unselected breast cancer cases and 4698 cancer-free women for the founder mutation. It was identified in 82 of 14,804 (0.55%) unselected cases and in 26 of 4698 (0.55%) controls (OR = 1.0; 95%CI 0.6–1.6). Clinical characteristics of breast cancers in the *BLM* mutation carriers and non-carriers were similar. Loss of the wild-type *BLM* allele was not detected in cancers from the *BLM* mutation carriers. No cancer type was more common in the relatives of mutation carriers compared to relatives of non-carriers. The *BLM* founder mutation p.Gln548Ter, which in a homozygous state is a cause of Bloom syndrome, does not appear to predispose to breast cancer in a heterozygous state. The finding casts doubt on the designation of *BLM* as an autosomal dominant breast cancer susceptibility gene.

## 1. Introduction

Homozygous mutations of the *BLM* gene are the cause of a rare recessive genetic disorder, Bloom syndrome, which is characterized by chromosomal instability, immunodeficiency, and a predisposition to different malignancies, including breast cancer [1]. The disease is most common in the Ashkenazi Jewish population but is represented (less frequently) in several Eastern European countries as well [2].

Human BLM protein belongs to the RecQ helicase family [3]. BLM plays many different functions in maintenance of genomic integrity [4]. For example, it is required for fork stability during unperturbed DNA replication. It stabilizes forks challenged by DNA damage or other agents that cause polymerase stalling and assists in replication restart [5]. BLM also plays an important role in homologous recombination DNA repair [6]. It senses DNA damage and recruits other repair proteins to the site of DNA breaks after irradiation in an ATM-dependent manner [7]. BLM is part of the BRCA1 multi-subunit protein complex, referred to as the BRCA1-genome surveillance complex, which includes other DNA damage repair proteins such as MSH2-MSH6 and MLH1, as well as ATM, NBS1, and MRE11 [8]. BLM has been implicated in the Fanconi anemia pathway through interaction with RAD51, RAD51D, and FANCJ [9]. Cells homozygous for a *BLM* mutation (from patients with Bloom syndrome) exhibit chromosomal instability characterized by a high rate of sister chromatid exchanges and vast structural rearrangements [10]. It is therefore not surprising that cancer is the most frequent complication in individuals with Bloom syndrome. Patients with Bloom syndrome are at high risk of a wide variety of cancer types. The distribution of cancers in individuals with Bloom syndrome is similar to that seen in the general population, but they occur at young age and multiple primary cancers are more common. There is clear evidence that *BLM* mutations in a homozygous state predispose to early onset breast cancer with a mean age of diagnosis of about 33 years [11]. Therefore, we reasoned that *BLM* is a good candidate for an autosomal dominant breast cancer susceptibility gene.

It is not clear if heterozygous carriers of *BLM* mutations are at increased cancer risk, and if so, whether *BLM* should be included in cancer test panels for clinical use. There are several association studies with inconsistent results. In the first study, Sokolenko et al. detected the c.1642C>T (p.Gln548Ter) mutation of *BLM* in 17 of 1498 Russian breast cancer cases and 2 of 1093 controls (OR = 6.3, *p* = 0.01) [12]. In the second study, Prokofyeva et al. reported the same mutation in 15 of 3188 cases and 2 of 2458 controls from Russia and Belarus (OR = 5.1, *p* = 0.03) [13]. In a later study, the p.Gln548Ter mutation was detected with equal frequency (0.4%) in breast cancer cases (6 of 1400) and in population controls from Russia (35 of 7920) (OR = 0.97, 95%CI 0.37–2.41, *p* = 0.9) [14].

The *BLM* p.Gln548Ter mutation is the most common cause of Bloom syndrome in Slavic individuals, including Poland and Russia [15]. Among Jews, the predominant mutation, referred to as BLMAsh is a 6-bp deletion and 7-bp insertion at nucleotide position 2207 of the *BLM* gene [2]. Two association studies of this mutation in Jewish women, by Gruber et al. and by Cleary et al., reported elevated but non-significant odds ratios of 1.8 (95%CI 0.6–4.9) and 1.6 (95%CI 0.5–5.4) respectively, for breast cancer, given the BLMAsh founder mutation [16,17]. In a cross-sectional study, no cancer type was found to be overrepresented among 326 relatives of BLMAsh mutation carriers, compared to 503 family members of controls [18]. In a prospective study of 152 women with the BLMAsh mutation, no cancer site was seen more often than expected (mean follow-up 10 years) [19]. In summary, the epidemiology evidence from Eastern Europe favored the hypothesis that *BLM* mutations play a role in breast cancer predisposition, but other studies have led us to question this hypothesis. 

To investigate whether or not the presence of a *BLM* mutation increases breast cancer risk, we studied approximately 15,000 women with breast cancer and 5000 controls from Poland. Further, we compared the clinical characteristics of breast cancers in carriers of a *BLM* mutation with cancers in non-carriers. We reviewed the pedigrees of women who have breast cancer and carry a *BLM* mutation and compared these with the pedigrees of breast cancer cases without a mutation. We analyzed loss of heterozygosity (LOH) at the *BLM* locus in breast cancer tissues from six women who carried a *BLM* truncating mutation.

## 2. Results

We identified a truncating mutation of the *BLM* gene in three of 617 women with hereditary breast cancer who underwent full gene sequencing. All three had the same mutation c.1642 C*>*T (p.Gln548Ter). This mutation in its homozygous state is the cause of Bloom syndrome [15,20].

In the second step, the c.1642 C*>*T (p.Gln548Ter) allele was genotyped in 14,804 patients with unselected breast cancer and 4698 controls. It was detected in 82 (0.55%) unselected cases and in 26 (0.55%) controls (OR = 1.0, 95%CI 0.6–1.6, *p* = 1.0). The mutation frequency in 2245 women with familial breast cancer (from unselected cases) was 0.49% (OR = 0.9, 95%CI 0.4–1.8, *p* = 0.9). The mutation frequency was 0.60% for women diagnosed at age of 50 years or below and was 0.50% for those diagnosed above the age of 50 years (Table 1).

The clinical characteristics of the patients with breast cancer with and without a *BLM* mutation is shown in Table 2. Carriers and non-carriers were similar in regards to age of diagnosis, histology, tumor size, lymph–node status and ER, PR, HER2 status. Bilateral tumors were similarly frequent in both groups (6.3% versus 4.7%; *p* = 0.7).

Survival data were available for 13,721 women with breast cancer. The mean follow-up time was 64 months. There were 15 deaths recorded in 81 *BLM* mutation carriers (18.5%) compared with 2368 deaths in 13,640 non-carriers (17.4%) (HR = 1.01, 95%CI 0.6–1.7, *p* = 0.98; log rank test). The 10-year survival was 76% for the carriers compared to 77% for non-carriers. After adjusting for age of diagnosis, the HR for mortality associated with the *BLM* mutation was 0.99 (95%CI 0.6–1.6; *p* = 0.97; Cox regression analysis).

We reviewed the pedigrees of women who carry the *BLM* mutation to see if there might be an excess of cancers at any site in first- or second-degree relatives. There were 63 cancers in 75 families with a *BLM* mutation (84%) versus 11,511 cancers in 13,556 *BLM* mutation negative families (85%) (Table 3). No particular cancer type was more common in the relatives of mutation carriers compared to relatives of non-carriers.

**Figure 1:**
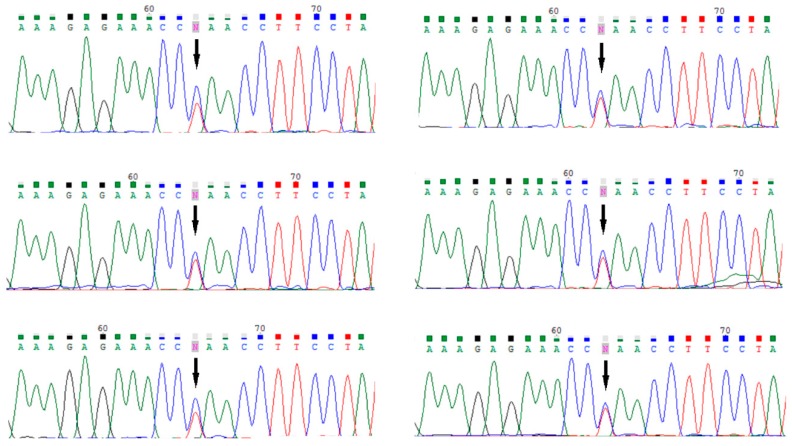
Loss of heterozygosity (LOH) analysis in breast cancer tissues from six carriers of *BLM* mutation; retention of the wild type *BLM* allele in breast cancer seen in six tested tumor samples. The p.Gln548Ter *BLM* mutation is indicated by arrow (↓).

We analyzed tumor DNA of six women with a *BLM* mutation for loss of heterozygosity. The wild-type *BLM* allele was retained in all cases (Figure 1).

## 3. Discussion

This is the largest study to date to evaluate the association between (heterozygous) mutations in the *BLM* gene and breast cancer. We approached the question in several ways, including a case-control study (association study) and a pedigree analysis. We also asked if cancers with and without *BLM* mutations showed differences in their clinical presentation and survival. Finally, we sought loss of heterozygosity at the *BLM* locus in tumor tissues (given that LOH is a signature event in most cancer syndromes with homologous repair deficiency). In none of these separate evaluations was there evidence of cancer predisposition for carriers of a *BLM* pathogenic mutation. 

We identified one recurrent truncating mutation of *BLM* (c.1642 C>T, p.Gln548Ter). This mutation is a cause of Bloom syndrome, as we and others diagnosed patients with Bloom syndrome homozygous for this protein-truncating variant of *BLM* [15,20]. However, this specific mutation appears to not be associated with an increased risk of breast cancer among Polish women. Our results contrast with the results of Sokolenko et al. and of Prokofyeva, et al., who reported separately that carriers of c.1642 C>T (p.Gln548Ter) founder mutation are at about a 6-fold increased risk of breast cancer [12,13]. Other studies showed no significant association and are in line with our negative study [14,16,17,18,19,20]. Our study is large and benefits from genetic homogeneity of Poland, which is populated by ethnic Slavs. The country is well-suited for association studies because the genetic similarity between cases and controls is assured, and spurious results due to admixture have not been reported. Patients with breast cancer were not selected for family history. 

There are several possible reasons for discrepancy between the results of our study and those of Sokolenko, et al., and of Prokofyeva, et al. It is possible that the differences are due to chance. It is not surprising that the first paper reported a positive association because of the tendency to submit positive results by authors and to accept positive results by journals. This is why it is essential that these studies be replicated prior to clinical implementation. It is not possible that differences between countries were due to different mutations as the same Slavic *BLM* founder mutation was studied. In our study we identified 108 carriers of the mutation in total, compared to a total of 36 for the first two positive studies combined. All subsequent studies have been negative [14,16,17,18,19,20]. Based on our study and the studies published to date, there is insufficient evidence that *BLM* is an autosomal dominant breast cancer susceptibility gene. 

The DNA damage signaling pathway plays a crucial role in the maintenance of the integrity of the genome in response to DNA damage and has been implicated in the pathogenesis of cancer. The gene from Bloom syndrome acts in the DNA damage repair signaling pathway [8]. It is interesting that heterozygous mutations in other genes in this pathway (i.e., *BRCA1, BRCA2*, *CHEK2*, *NBN, ATM)* predispose to breast cancer, but a heterozygous mutation in *BLM* does not appear to be pathogenic for breast cancer. 

*BRCA1, BRCA2* and *NBN* act as classical tumor suppressor genes. Both alleles of these tumor suppressor genes are inactivated before tumor formation [21]. LOH is observed in most cancers in *BRCA1, BRCA2*, and *NBN* carriers [22,23]. However, for some genes and cancer types, loss or mutation of a single allele may be sufficient to promote tumorigenesis. This phenomenon may be caused by a gene-dosage effect (haploinsufficiency) or the inactivating property the mutant protein may have on a particular pathway (dominant-negative effect) [24]. For example, it has been shown that the mechanism of tumorigenesis in association with *CHEK2* variants does not involve LOH. It is proposed that the mechanism by which some CHEK2 mutants may contribute to tumorigenesis is haploinsufficiency, i.e., due to lower level of expression [25,26,27]. Similarly, tumors that develop in heterozygous carriers of *ATM* missense mutations appear to be the consequence of a dominant-negative effect of the ATM protein [28,29]. As expected from the dominant-negative model, only mutations that do not lead to the absence of protein appear to be pathogenic (i.e., missense mutations or in-frame deletions). Some missense mutations in *CHEK2* may also have a dominant-negative effect. For example, CHEK2 I157T protein is stable and it dimerizes with the wild-type CHEK2 co-expressed in human cells. This variant may have negative effect on the pool of normal CHEK2 protein in heterozygous carrier cells by formation of heterodimers with wild-type CHEK2 [30]. 

Our robust analysis suggests that carriers of a heterozygous mutation in *BLM* are not at elevated risk of breast cancer. It has been reported that tumors in *BLM* mutation carriers do not demonstrate LOH, and normal BLM protein is expressed in tumor cells [16,22,31]. In our study, LOH analysis at the *BLM* locus was performed in DNA isolated from breast cancers from six *BLM* (p.Gln548Ter) mutation-positive women. The wild-type *BLM* allele was retained in all cases. It is therefore possible that loss of function of a single *BLM* allele (i.e., haploinsufficiency) is not sufficient to promote carcinogenesis in the breast.

## 4. Material and methods

### 4.1. Hereditary Breast Cancer Cases

For the first step, we selected 617 unrelated breast cancer patients from 617 Polish families with a family history of breast cancer. We included women with a strong family history for breast cancer (in first- and second-degree relatives). Among the 617 probands with breast cancer, there were 160 women from families with at least four women affected with breast cancer, 378 women from families with three affected, and 79 women from families with two affected (at least one had bilateral breast cancer or breast cancer below age 50). The mean number of breast cancers per family was 3.4. Among the 617 probands with breast cancer, 104 women were diagnosed at age 40 years or below, 226 women were diagnosed at age 41 to 50 years, 200 women were diagnosed at age 51 to 60 years, and 87 women were diagnosed above age of 60 years. The mean age of breast cancer diagnosis among the 617 probands was 46 years (range 28 to 76 years). Of the 617 patients, 81 reported a family history of ovarian cancer and 16 reported a family history of male breast cancer. The 617 probands were selected from a registry of 3,519 familial breast cancer cases housed at the Hereditary Cancer Center in Szczecin based on the number and age of onset of breast cancer cases among their relatives, and based on that they tested negative for a panel of 17 founder Polish mutations of *BRCA1, BRCA2, CHEK2, PALB2, NBN*, and *RECQL* [32]. All probands were ethnic Poles. 

### 4.2. Unselected Cases of Breast Cancer

We studied 14,804 prospectively ascertained cases of invasive breast cancer, diagnosed from 1996 to 2012, at 18 different hospitals in Poland (mean age of diagnosis 54 years, range 18–93). All women who were diagnosed with a first primary invasive breast cancer at the participating centers were eligible. Patients with purely intraductal or intralobular cancer were excluded (DCIS or LCIS) but patients with DCIS with micro-invasion were included. Patients were unselected for family history. The patient participation rate among invited women was 76.1%. Information was recorded on clinical characteristics of breast cancers through review of medical records. Family history included the number of first- and second-degree relatives with any cancer. Of the 14,804 unselected cases, 2245 patients (16%) reported at least one first- or second-degree relative with breast cancer (familial breast cancer cases from unselected series). The definition of a familial case (from unselected cases) was the presence of one or more breast cancers in first or second degree relatives of a womanwith unselected breast cancer (i.e., two or more breast cancers in first or second degree relatives in a family). Survival data were obtained (status: alive or dead, the date of death) from Polish Ministry of the Interior and Administration in July 2014. The Ethics Committee of Pomeranian Medical University in Szczecin approved the study (IRB No. KB-0012/97/17). 

### 4.3. Controls

The purpose of the control group was to estimate the *BLM* mutation frequency in the underlying Polish population. The control group included 4,698 Polish cancer-free women age 18 to 94 years (mean age, 53.0 years) derived from four sources. The first consisted of 987 women from the region of Szczecin (age range, 24 to 84 years) who were part of a population-based study of the 1.5 million residents of West Pomerania (North-West Poland) designed to identify familial aggregations of cancer and were interviewed in 2007. The second series consisted of 1717 unselected women (age range 32–72) who participated in mammography screening at 8 different centers all over Poland between 2009 and 2011 (Kielce, Legnica, Olsztyn, Poznań, Szczecin, Świdnica, Toruń, and Zielona Góra) and provided a blood sample for DNA analysis. The third control group included 1036 women (age range, 20–94 years) selected at random from the computerized patient lists of family practices located in the region of Opole (South Poland). These women were invited to participate by mail and participated in 2012 and 2013. The fourth series included 958 Polish women (age range 50–66 years), who participated in a colonoscopy screening program for colorectal cancer between 2007 and 2010 in Szczecin, Białystok and Lódź [33]. 

### 4.4. Sequencing of the BLM Gene

We analyzed the entire coding sequence of *BLM* from the exome sequencing data of 617 women with hereditary breast cancer (step 1) as described previously [33]. The Agilent SureSelect human exome kit (V6) was used for capturing target regions. The regions were sequenced on Illumina NextSeq 500. The mean depth of coverage was approximately 100×, 97.4% of the CCDS exons were covered at 20× depth of coverage and higher, which was used for variant calling. We looked for protein truncating genetic variants, including frame shift insertions, deletions, stop codon mutations, and variants at the consensus splice sites, which are likely to be dysfunctional.

### 4.5. Genotyping

We genotyped 14,804 women with breast cancer and 4,698 controls for the *BLM* mutation, which was detected by sequencing (step 2). DNA was isolated from 5 to 10 mL of peripheral blood. The c.1642C>T (p.Gln548Ter) mutation was genotyped using a TaqMan assay (Thermo Fisher Scientific, Waltham, MA, USA) in a LightCycler Real-Time PCR 480 System (Roche Life Science, Mannheim, Germany). All mutations were confirmed by Sanger sequencing. Sequencing reactions were performed using a BigDye Terminator v3.1 Cycle Sequencing Kit (Thermo Fisher Scientific) according to the manufacturer’s protocol. Sequencing products were analyzed on the ABI prism 3100 Genetic Analyzer (Thermo Fisher Scientific).

### 4.6. Loss of Heterozygosity Analysis

Formalin-fixed paraffin-embedded (FFPE) tissue samples from eight *BLM* mutation carriers were available in the Department of Pathology of Pomeranian Medical University in Szczecin. A pathology review of these samples was conducted by a pathologist associated with the study. Tissue samples of good quality were available from six patients. Loss of heterozygosity (LOH) analysis at the *BLM* locus was performed in DNA from micro-dissected tumors from six *BLM* mutation-positive women (p.Gln548Ter) using methodology described previously [34] with minor modifications: (1) DNA was isolated with QIAamp DNA FFPE Tissue Kit (from QIAGEN); (2) LOH was analyzed by direct Sanger sequencing of a 155 bp DNA fragment containing the p.Gln548Ter mutation (forward primer 5′ ctcttatttcccaggaaatgttctc; reverse primer 5′ cttcccagtcatcatcatcatc). 

### 4.7. Statistical Analysis

The prevalence of the *BLM* allele p.Gln548Ter was estimated in 14,804 breast cancer cases and 4,698 cancer-free women. Odds ratios were generated from two-by-two tables. Women with breast cancer, with and without a *BLM* mutation, were compared for age at diagnosis and clinical features of the breast cancers. Statistical significance was assessed using Fisher exact test or Chi-squared test where appropriate. Means were compared using t-test. 

To estimate the survival of women with and without the mutation, we followed the breast cancer patients from the date of diagnosis until the date of death or July 2014. We compared the survival between mutation carriers and non-carriers by log-rank test. An age-adjusted hazard ratio was calculated using Cox regression analysis.

## 5. Conclusions

Our study and the studies published to date do not support the hypothesis that *BLM* mutations, in a heterozygous state, confer elevated risk of breast cancer (and probably of other cancers). We conclude that the *BLM* gene should not be included in cancer test panels for clinical use. Women with heterozygous *BLM* mutations should not be advised that they are at increased risk of breast cancer, and should not be counseled to intensify surveillance. 

## Figures and Tables

**Table 1:** Prevalence of *BLM* p.Gln548Ter founder mutation in 14,804 women with breast cancer, by age and family history and in 4,698 cancer-free women.

Group	Total (*n*)	*BLM* p.Gln548Ter Mutation Positive	Prevalence (%)	OR (CI 95%)	*p*-Value
**Patients with Breast Cancer**					
All cases	14,804	82	0.55%	1.0 (0.6–1.6)	1.0
**Age (years)**					
≤40	1791	14	0.78%	1.4 (0.7–2.7)	0.4
41–50	6145	34	0.55%	1.0 (0.6–1.7)	1.0
51–60	3289	16	0.49%	0.9 (0.5–1.6)	0.8
61–70	2247	12	0.53%	1.0 (0.5–1.9)	0.9
≥71	1332	6	0.45%	0.8 (0.3–2.0)	0.8
Number of Relatives with Breast Cancer *					
0	11,387	64	0.56%	1.0 (0.6–1,6)	0.9
1	1719	8	0.47%	0.8 (0.4–1.9)	0.8
≥2	526	3	0.57%	1.0 (0.3–3.4)	1.0
**Reference**					
Cancer-free controls	4698	26	0.55%	-	-

Footnote: Odds ratios and *p* values calculated using cancer-free controls as a reference group. * Refers to first-degree or second-degree relatives.

**Table 2:** Clinical characteristics of breast cancers in carriers of the *BLM* p.Gln548Ter mutation and non-carriers.

Characteristic	*BLM* p.Gln548Ter Positive Cases *n* = 82	*BLM* p.Gln548Ter Negative Cases *n*= 14,722	*p*-Value
**Age at diagnosis (years)**	52.8 (29 – 79)	53.7 (18–93)	0.5
**Histological features**			
Ductal, grade 3	11/59 (18.6%)	2409/11,560 (20.8%)	0.8
Ductal, grade 1–2	25/59 (42.4%)	4724/11,560 (40.1%)	1.0
Ductal, grade unknown	7/59 (11.9%)	858/11,560 (7.4%)	0.3
Medullary	4/59 (6.8%)	393/11,560 (3.4%)	0.3
Lobular	7/59 (11.9%)	1502/11,560 (13.0%)	0.9
Tubulolobular	0/59 (0%)	156/11,560 (1.3%)	0.7
DCIS with microinvasion	0/59 (0%)	410/11,560 (3.5%)	0.3
Other or undefined	5/59 (8.5%)	1108/11,560 (9.6%)	0.9
**Receptor status**			
Oestrogen receptor-positive	34/52 (65.4%)	7004/10,372 (67.5%)	0.9
Progesterone receptor-positive	35/49 (71.4%)	6959/9891 (70.4%)	1.0
HER2-positive	9/42 (21.4%)	1520/8392 (18.1%)	0.7
Triple-negative	9/42 (21.4%)	1387/8004 (17.3%)	0.6
**Size (cm)**			
<1	3/47 (6.4%)	1041/9621 (10.8%)	0.5
1–1,9	20/47 (42.6%)	3882/9621 (40.3%)	0.9
2–4,9	22/47 (46.8%)	4272/9621 (44.4%)	0.9
≥5	2/47 (4.3%)	426/9621 (4.4%)	1.0
**Lymph node-positive**	25/51 (49.0%)	4423/9883 (44.8%)	0.6
**Bilateral**	4/63 (6.3%)	563/11,984 (4.7%)	0.7
**Chemotherapy (yes)**	41/63 (65.1%)	6499/10,680 (60.9%)	0.6
**Tamoxifen (yes)**	26/42 (61.9%)	5228/7969 (65.6%)	0.7
**Vital status (deceased)**	15/81 (18.5%)	2369/13,640 (17.4%)	0.9

Footnote: Data are mean (range) or number/total (%), *p*-value compares mutation-positive with mutation-negative patients and was calculated with Fisher’s exact test, DCIS = ductal carcinoma in situ.

**Table 3:** Cancers reported in the families of the 75 unselected breast cancer cases of a *BLM* mutation compared to those reported by the 13,557 non-carrier cases.

Cancer Site	Number (%) of Cancers in Relatives of *BLM* p.Gln548Ter Positive Women (*n* = 75 families)		Number (%) of Cancers in Relatives of *BLM* p.Gln548Ter Negative Women (*n*= 13,557 families)		*p*-Value
	*n*	%	*n*	%	
**Breast**	11	14.7%	2234	16.5%	0.8
**Colon**	6	8.0%	1041	7.7%	0.9
**Kidney**	3	4.0%	376	2.8%	0.8
**Larynx**	0	0.0%	525	3.9%	0.2
**Lung**	14	18.7%	2007	14.8%	0.4
**Leukemia or Lymphoma**	3	4.0%	525	3.9%	0.9
**Pancreas**	2	2.7%	390	2.9%	0.9
**Prostate**	6	8.0%	909	6.7%	0.8
**Stomach**	6	8.0%	1161	8.6%	0.9
**Cervix or Endometrium**	9	12.0%	1408	10.4%	0.8
**Ovary**	3	4.0%	479	3.5%	0.8
**All cancers**	63	84.0%	11,511	84.9%	0.9

Footnote: family history in first- and second-degree relatives was available for 75 of 82 (91%) *BLM* mutation carriers and 13,557 of 14,722 (92%) non-carriers.

## Appendix A

Other members of Polish Hereditary Breast Cancer Consortium are: M. Bębenek, D. Godlewski, S. Gozdecka-Grodecka, S. Goźdź, O. Haus, H. Janiszewska, M. Jasiówka, E. Kilar, R. Kordek, B. Kozak-Klonowska, G. Książkiewicz, A. Mackiewicz, E. Marczak, J. Mituś, Z. Morawiec, S. Niepsuj, R. Sibilski, M. Siołek, J. Sir, D. Surdyka, A. Synowiec, C. Szczylik, R. Uciński, B. Waśko, R. Wiśniowski, T. Byrski, B. Górski.
